# Tophaceous Gout

**DOI:** 10.1002/ccr3.70262

**Published:** 2025-02-20

**Authors:** Yunxin Liu, Yang Jiao

**Affiliations:** ^1^ Department of General Practice (General Internal Medicine) Peking Union Medical College Hospital, Chinese Academy of Medical Sciences & Peking Union Medical College Beijing China

**Keywords:** arthritis, gout, hyperuricemia, overhanging edges, tophus

## Abstract

Chronic untreated hyperuricemia can lead to polyarticular tophaceous gout from recurrent arthritis, which could cause bone erosion and joint damage. “Overhanging edges” on X‐ray, indicative of bony erosions, are highly specific for gout. Tophaceous gout, though more common in older adults, can occur in younger individuals with prolonged hyperuricemia.

## Graphical abstract

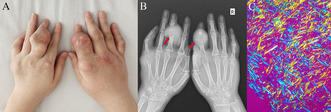

A 32‐year‐old man presented with a 10‐year history of hyperuricemia, but he had refused uric acid‐lowering treatment. Over the past 2 years, multiple subcutaneous nodules had developed over his hand and foot joints accompanied by intermittent acute joint pain that was relieved by non‐steroidal anti‐inflammatory drugs. On admission, physical examination showed multiple tophi and joint deformities in both hands (Figure 1A) and feet. His serum uric acid level was 559 μmol/L and creatinine was normal. Hand X‐rays revealed bony destruction with delicate overhanging edges (Figure 1B) in the affected hand joints. Arthrocentesis was conducted and monosodium urate crystals were identified (Figure 1C). The diagnosis of gout was confirmed. He was treated with allopurinol 400 mg/day. His serum urate remained at 330 μmol/L a month later and there have been no repeat episodes of acute arthritis after half a year follow‐up.

**FIGURE 1:**
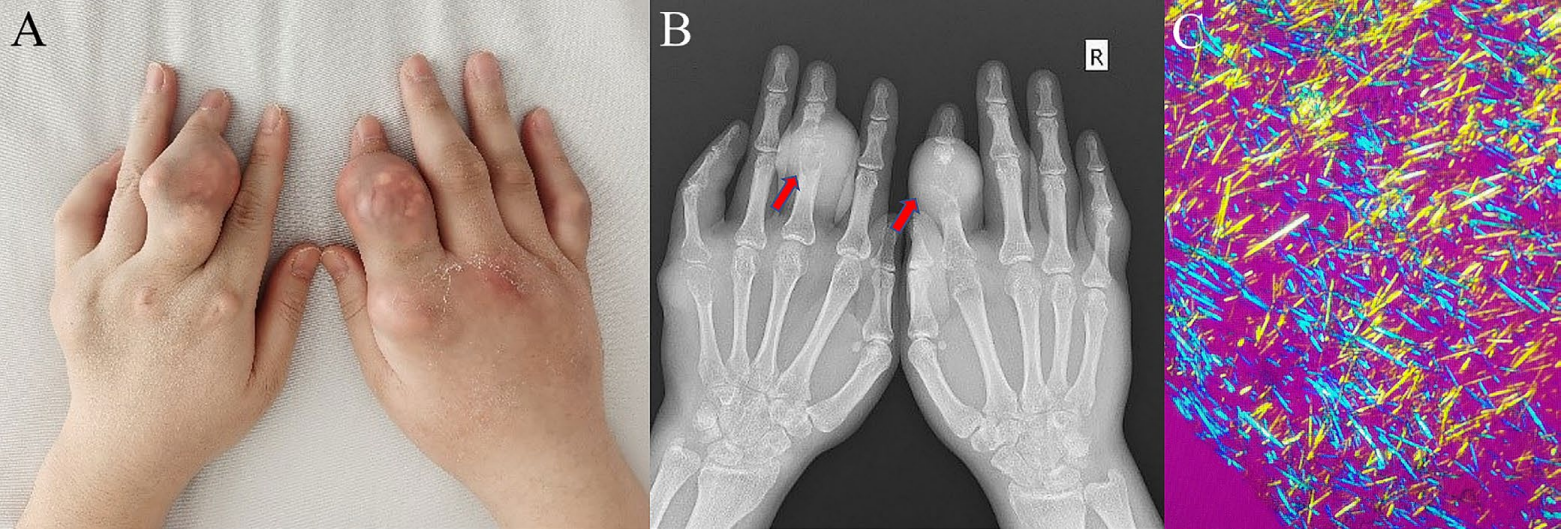
(A) Physical examination showed gout tophi at multiple joints in the hands. (B) Hand X‐ray revealed multiple destructive erosions of the bones and soft tissue swelling with significant delicate overhanging edges at the left third and right second proximal interphalangeal joints (red arrows). (C) Synovial fluid revealed needle‐shaped, negatively birefringent crystals under polarized light microscopy.

Tophaceous gout is formed due to the deposition of monosodium urate crystals in the intra‐ and extra‐articular soft tissues and bones [1]. Tophi presenting on the ear or in the soft tissues around the joints may provide strong clues to the diagnosis, while overhanging edges of bone associated with bony erosions on plain radiography are also specific to gouty lesions. Untreated gout could lead to the development of bone erosion and joint damage. Although tophaceous gout is more common in older adults, it can also affect younger individuals and should be considered in the differential diagnosis of arthritis in this population. Early diagnosis and treatment with uric acid‐lowering therapy are crucial to preventing disease progression and managing symptoms effectively.

## Author Contributions

**Yunxin Liu:** data curation, formal analysis, writing – original draft. **Yang Jiao:** conceptualization, formal analysis, writing – review and editing.

## Ethics Statement

Our study has been granted an exemption from the review by the Institutional Review Board of Peking Union Medical College Hospital.

## Consent

Written informed consent was obtained from the patient for the publication of this case report and any accompanying images.

## Conflicts of Interest

The authors declare no conflicts of interest.

## Data Availability

The data that support the findings of this study are available from the corresponding author upon reasonable request.
